# Imaging Pulmonary NF-kappaB Activation and Therapeutic Effects of MLN120B and TDZD-8

**DOI:** 10.1371/journal.pone.0025093

**Published:** 2011-09-22

**Authors:** Dan Ansaldi, Eldad A. Hod, Fabio Stellari, Jae-Beom Kim, Ed Lim, Mark Roskey, Kevin P. Francis, Rajendra Singh, Ning Zhang

**Affiliations:** 1 Caliper Life Sciences, Alameda, California, United States of America; 2 Department of Pathology and Cell Biology, Columbia University College of Physicians and Surgeons, New York, New York, United States of America; 3 In Vivo Pulmonary Pharmacology Department, Chiesi Group, Parma, Italy; Vanderbilt University Medical Center, United States of America

## Abstract

NF-*κ*B activation is a critical signaling event in the inflammatory response and has been implicated in a number of pathological lung diseases. To enable the assessment of NF-*κ*B activity in the lungs, we transfected a luciferase based NF-*κ*B reporter into the lungs of mice or into Raw264.7 cells in culture. The transfected mice showed specific luciferase expression in the pulmonary tissues. Using these mouse models, we studied the kinetics of NF-*κ*B activation following exposure to lipopolysaccharide (LPS). The Raw264.7 cells expressed a dose-dependent increase in luciferase following exposure to LPS and the NF-*κ*B reporter mice expressed luciferase in the lungs following LPS challenge, establishing that bioluminescence imaging provides adequate sensitivity for tracking the NF-*κ*B activation pathway. Interventions affecting the NF-*κ*B pathway are promising clinical therapeutics, thus we further examined the effect of IKK-2 inhibition by MLN120B and glycogen synthase kinase 3 beta inhibition by TDZD-8 on NF-*κ*B activation. Pre-treatment with either MLN120B or TDZD-8 attenuated NF-*κ*B activation in the pulmonary tissues, which was accompanied with suppression of pro-inflammatory chemokine MIP-1ß and induction of anti-inflammatory cytokine IL-10. In summary, we have established an imaging based approach for non-invasive and longitudinal assessment of NF-*κ*B activation and regulation during acute lung injury. This approach will potentiate further studies on NF-*κ*B regulation under various inflammatory conditions.

## Introduction

Chronic exposure to significant levels of lipopolysaccharide (LPS), which is ubiquitously present as a contaminant on airborne particles [1], is reported to be associated with the development and progression of many types of lung diseases including asthma and chronic obstructive pulmonary disease (COPD) [2]. The pathogenesis of COPD is characterized by pulmonary inflammation associated with increased accumulation of neutrophils and pro-inflammatory cytokines in the bronchoalveolar lavage fluid [3]. In experimental animals, repeated intratracheal LPS instillation results in persistent chronic pulmonary inflammation with pathologic changes mimicking certain aspects of clinical COPD [4].

The NF-κB transcription factor consists of subunits that associate in dimers [5]. The commonly described dimer forms are p50 (NF-κB1) with RelA (p65) and p52 (NF-κB2) with RelB. The NF-κB1/RelA complex remains in the cytoplasm in an inactive form in association with IκBα. Upon stimulation with LPS or pro-inflammatory cytokines, IKK2 is activated and causes IκBα phosphorylation, leading to its ubiquitinization and proteasomal degradation. This releases the NF-κB1/RelA complex from IκBα inhibition and allows it to translocate to the nucleus where it transcriptionally activates many key genes involved in inflammation and chemotaxis [6]–[8]. Abnormal NF-κB activation has been implicated in the pathophysiology of COPD [9], thus NF-κB pathway blockers, such as MLN120B, are currently being explored for treatment of inflammatory diseases such as COPD and asthma [10].

Glycogen synthase kinase (GSK)-3β is a ubiquitous serine-threonine protein kinase involved in glycogen metabolism [11]. There are two mammalian isoforms of GSK-3, encoded by different homologous genes, GSK-3α and GSK-3β [12]. GSK-3β phosphorylates and regulates many substrates, including transcription factors such as β-catenin [13], NF-κB [14], STAT [15] and other substrates that are critical for metabolism, protein translation, cell cycle, oncogenesis, and neuroprotection [16]-[18]. Absence of GSK-3β severely affects the TNF-induced activation of NF-κB p65 [14] and potentiates TNF-induced apoptosis [19]. Thus, GSK-3β blockers, such as TDZD-8, have therapeutic potential for treating inflammatory respiratory diseases.

Animal models for assessing NF-κB activity non-invasively have been of interest for pre-clinical drug development. Luciferase reporter based animal models that used either short tandem repeat of NF-κB responsive elements or the promoters of NF-κB responsive genes have been developed [20]–[23]. Using several of these NF-κB reporter mice [20], [21], we attempted to image NF-κB activation in the lungs non-invasively and observed that a systemic response was induced in these animals even with local intratracheal delivery of LPS. The bioluminescent signal from superficial tissues interferes with accurate assessment of NF-κB activation in pulmonary tissues. Due to this limitation, we have explored a tissue specific gene delivery approach to generate mice with specific NF-κB-luciferase reporter expression in pulmonary tissues and validated the utility of non-invasive imaging approaches for assessing the efficacy of inhibitors of the NF-κB signaling pathway.

## Results

### In vitro comparison of NF-κB reporters

**Figure 1:**
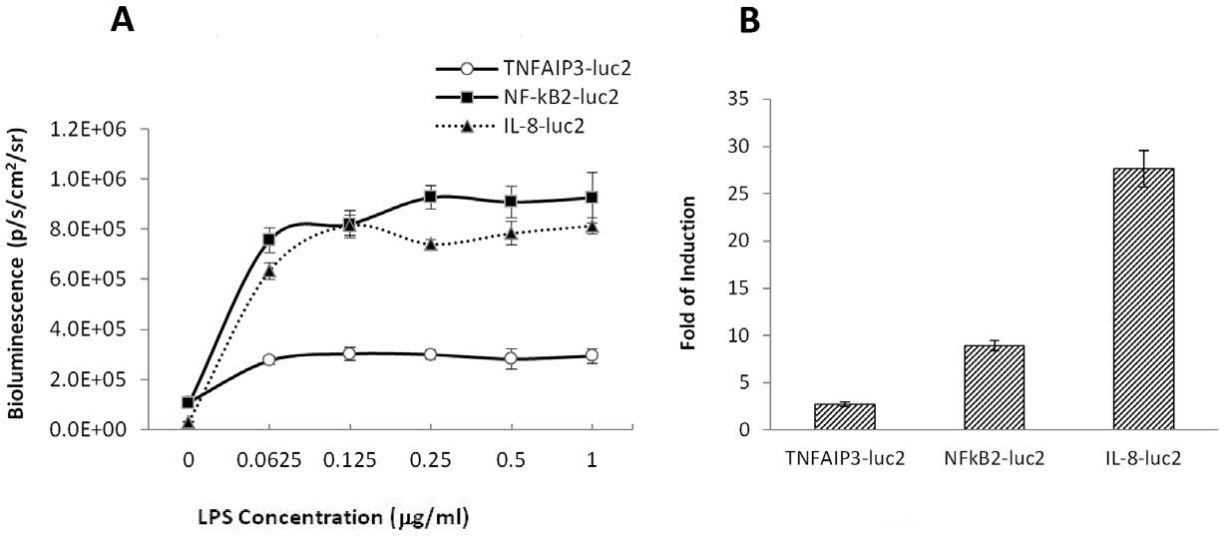
In vitro comparison of NF-κB reporters. RAW264.7 cells were transiently transfected with NF-κB reporters. Cells were treated with LPS at 0–1 µg/ml concentrations overnight and imaged with IVIS after adding luciferin. Quantification of luciferase signal (A). Fold change of cells treated with 1 µg/ml LPS compared to vehicle treated cells (B). Data presented as mean ± SEM; n = 4 wells per reporter construct and LPS concentration of 1 µg/ml.

We transiently transfected three NF-κB reporters into RAW264.7 cells and monitored their response to LPS treatment. All three reporters showed induction of luciferase expression after LPS treatment (Fig 1A). With 1 µg/ml of LPS, the IL8-luc2 and NF-κB2-luc2 reporters exhibited a 28- and 9-fold induction of luciferase, respectively, while the TNFAIP3-luc2 reporter showed a less robust induction of 3-fold under these same conditions (Fig 1B).

### In vivo gene delivery of NF-κB reporters to lung tissues

Incorporation of DNA into cells is influenced by the charge of the particle, with an overall positive charge required for DNA entry into cells. Since DNA is negatively charged, the use of JetPEI is needed to produce a cationic polyethylenimine (PEI)/DNA complex capable of incorporating into cells in vivo. The N/P ratio (Nitrogen/Phosphate ratio) of JetPEI used helps to determine the overall charge of the DNA complex. Complexes are electroneutral at an N/P ratio of 2–3, and the best transfections are achieved when using an N/P ratio of 5–8, depending on mouse strain used and target delivery area. We compared albino C57BL/6 and BALB/cJ mice in this gene delivery procedure and found that BALB/cJ mice were more susceptible to mortality than albino C57BL/6 mice. We observed a survival rate of 79% and 82% when the N/P ratio of JetPEI was 7 and 6, respectively, using albino C57BL/6 mice injected with 50 µg of DNA each. When BALB/cJ mice were used (n = 14) with 50 µg of DNA and an N/P ratio of 7, all mice died within 24 hours. Increased survival (2/2 mice) of BALB/cJ mice was observed when the DNA quantity was reduced to 25 µg and the N/P ratio reduced to 4.

**Figure 2:**
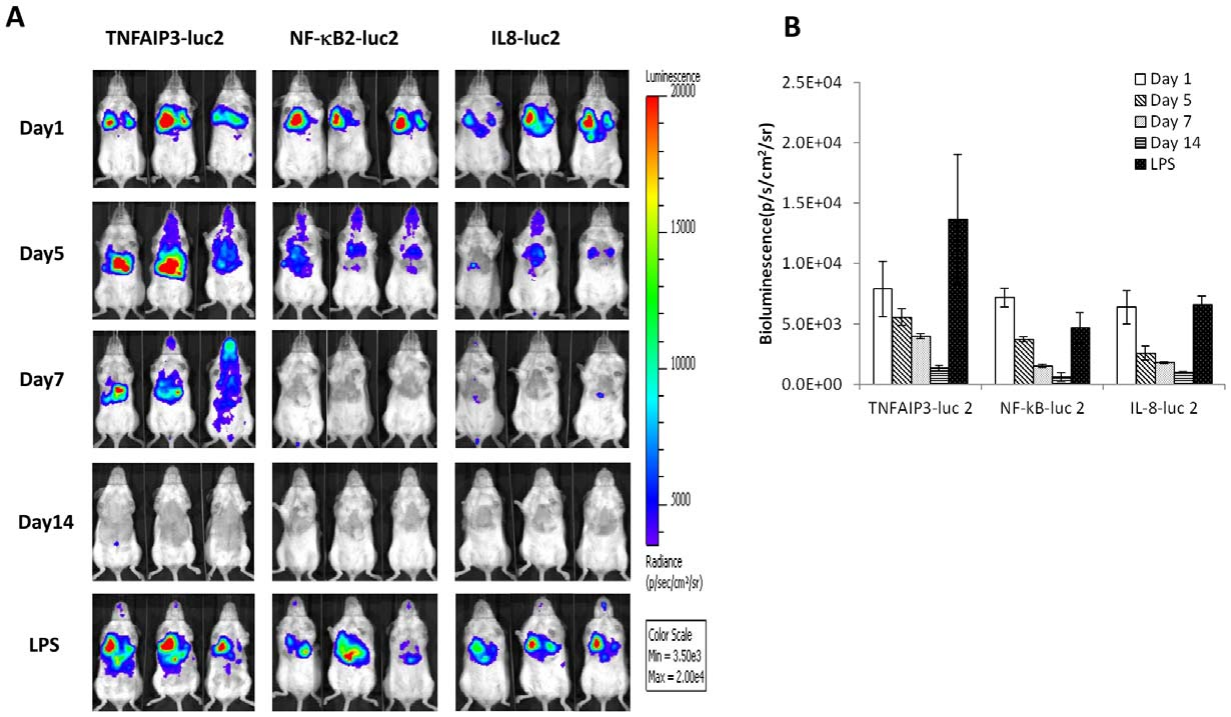
In vivo gene delivery of NF-κB reporters to the lung and response to LPS treatment. Albino C57BL/6 mice (n = 3 per group) were injected intravenously with NF-κB reporter DNA using JetPEI. Mice were imaged following i.p. injection of luciferin at 1, 5, 7, and 14 days following transfection. After the imaging on the day 14, mice were intratracheally challenged with LPS at 1 mg/kg and re-imaged after 3 hours. Bioluminescence images (A) and quantification of bioluminescence signal from the lungs (B). Quantification was performed by drawing a region of interest (ROI) over the chest area followed by quantification using the LivingImage software. Data presented as mean ± SEM.

We used in vivo-JetPEI to deliver the NF-κB reporters to the lung tissues as described above. Mice were imaged at multiple time points following DNA delivery (Fig 2A). After 1 day, mice injected with each of the three NF-κB reporters showed specific bioluminescence signals in the lungs, which gradually decreased over time. By 14 days following delivery, the expression decreased to a low basal level. We used these mice to assess their responsiveness to LPS treatment. Thus, at two weeks after DNA delivery, LPS was delivered into these mice intratracheally and they were imaged 3 hours later (Fig 2A). All three reporters showed induction of luciferase signal specifically in the lungs (Fig 2A,B) and the induction was 14.3-, 9.6-, and 6.6-fold for TNF-AIP3-luc2, NF-κB 2-luc2, and IL8-luc2 transfected mice, respectively.

### Kinetics of NF-κB activation following LPS and TNF-α challenge

**Figure 3:**
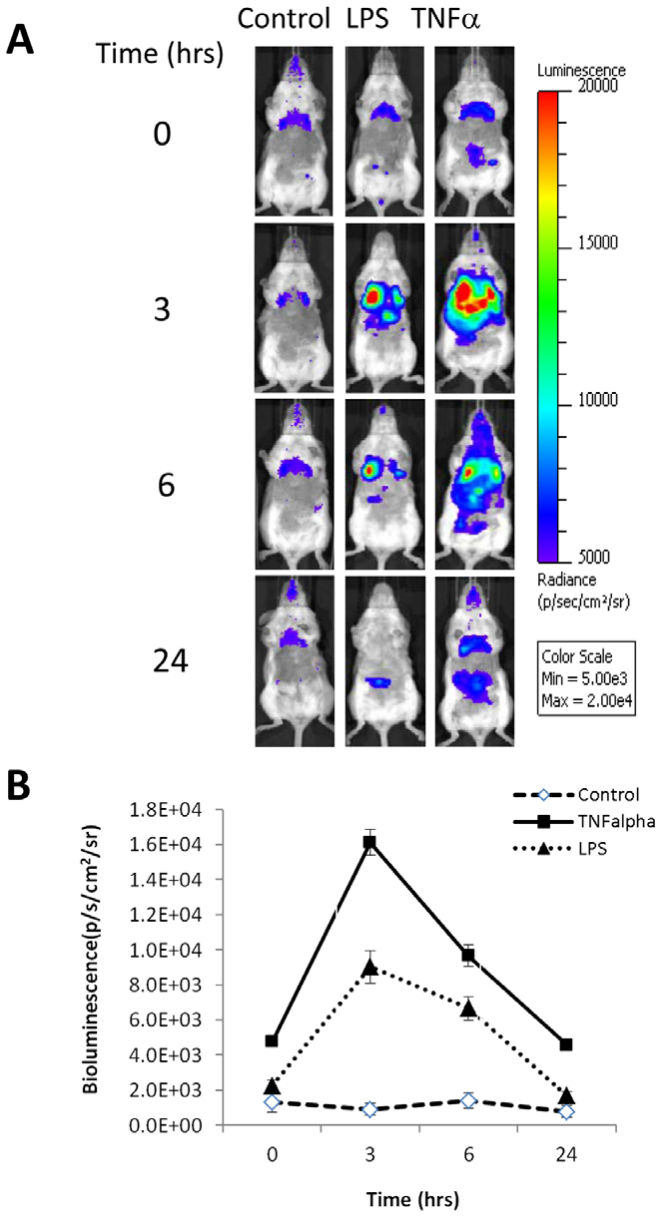
Kinetics of NF-κB activation. TNFAIP3-luc2 reporter transfected mice were challenged with LPS (1 mg/kg, intratracheally, n = 11) or TNFα (1 µg/mouse, intravenously, n = 4) or saline (n = 4). Mice were imaged at 0 (prior to injection), 3, 6, and 24 hours after injection. Bioluminescent images from one representative mouse per group (A) and quantification of lung signals (B) are shown. Data presented as mean ± SEM.

The kinetics of luciferase induction by LPS and TNFα was studied using TNFAIP3-luc2 transfected mice. LPS (1 mg/kg) was delivered intratracheally or TNFα was injected intravenously at a dose of 1 µg/mouse. Both LPS and TNFα induced a specific signal in the lungs 3 hours after injection (Fig 3A). Quantification of the lung signal shows that the induction reached a plateau at 3 hours following LPS or TNFα administration and declined at 6 hours. (Fig 3B). We also used TNFα at 0.5 and 2 µg/mouse and achieved a comparable level of luciferase induction in the lungs (data not shown). Similarly, LPS at 2 mg/kg induced a similar level of NF-κB activation as the 1 mg/kg dose (data not shown).

### Comparison of anti-inflammatory compounds on NF-κB induction in vitro

**Figure 4:**
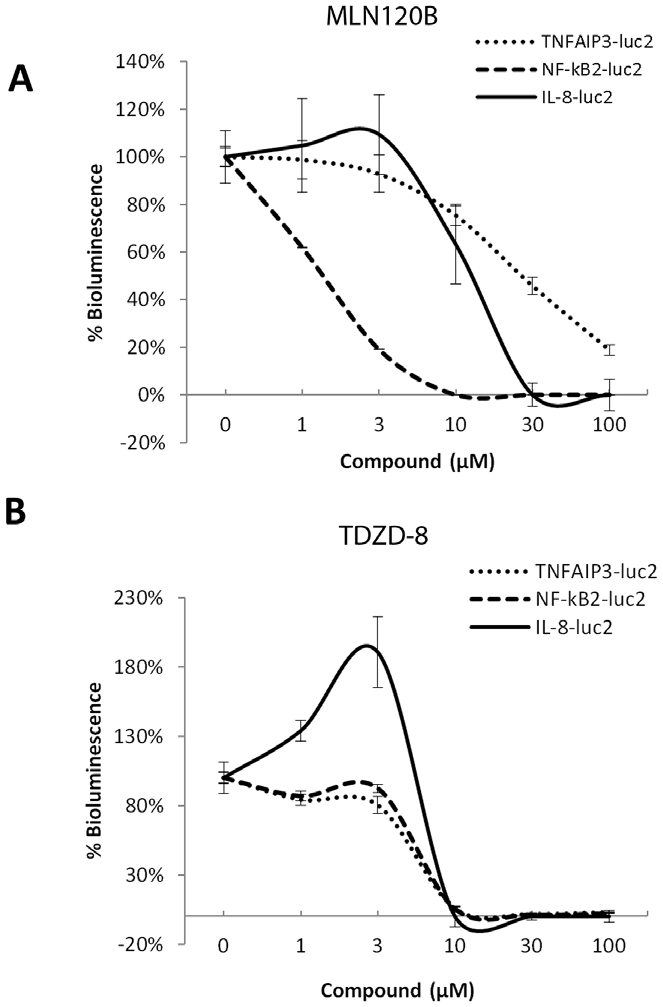
Effect of anti-inflammatory agents on NF-κB induction in vitro. The effect of MLN120B (A) and TDZD-8 (B) on luciferase induction by LPS in TNFAIP3-luc2, NF-κB2-luc2 and IL8-luc2 transfected RAW264.7 cells are shown. All the compounds were tested at 1–100 µM concentrations. The data are presented as percentage change over vehicle treated cells. Data presented as mean ± SEM; n = 4 wells per reporter construct and LPS concentration.

Two anti-inflammatory compounds: MLN120B, an IKK2 inhibitor; and TDZD-8, a GSK-3β inhibitor, were compared for their effects on LPS induced NF-κB activation. We transiently transfected the NF-κB reporters into RAW267.4 cells and induced inflammation in the cells with LPS. The effect of these compounds on LPS induced luciferase expression was assayed. Both compounds showed an inhibitory effect on LPS induced NF-κB activation (Fig 4). The IC50 value of MLN120B was 1.4, 14.8 or 27.3 µM for NF-κB2-luc2, IL8-luc2 or TNF-AIP3-luc2 reporter transfected cells, respectively, indicating that IKK2 inhibition affected the transcriptional activity of the NF-κB2 promoter most effectively. Treatment with TDZD-8 resulted in a much more uniform inhibition of the three NF-κB reporters with IC50 values for TNF-AIP3-luc2, NF-κB2-luc2, or IL8-luc2 of 4.0, 6.9, or 8.4 µM, respectively.

### IKK2 inhibition attenuates NF-κB activation in the lungs

**Figure 5:**
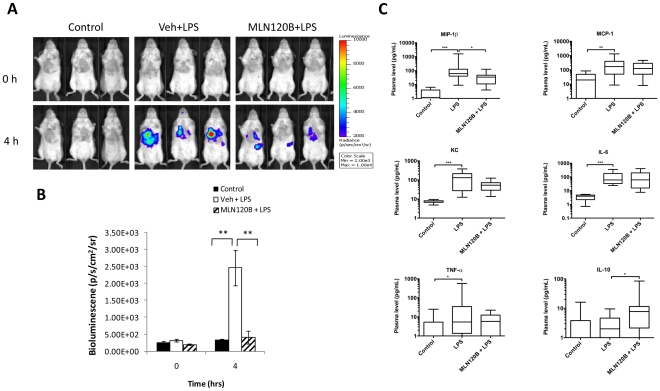
Effect of IKK2 inhibition on NF-κB induction in vivo. At 2 weeks after in vivo gene delivery with the NF-κB2-luc2 reporter, mice (n = 7) were pre-treated with MLN120B at 300 mg/kg orally or vehicle control 16 and 1-hour prior to LPS delivery. Mice were imaged immediately before LPS injection (T = 0) and at 4 hours. Bioluminescence images (A) and quantification of lung signals (B) are shown. Cytokines measured in bronchial lavage fluid as specified (C). Data presented as standard box and whisker plots, *p<0.05, **p<0.01, ***p<0.001 by Mann-Whitney U test.

Using NF-κB reporter transfected mice, we tested the effect of the IKK2 inhibitor, MLN120B, on LPS induced NF-κB activation in the lungs. This compound failed to show any effect when TNFAIP3-luc2 transfected mice were used (data not shown). Since the TNFAIP3 gene is involved in the negative feedback loop of NF-κB signaling [25], the luciferase signal on the one hand may represent NF-κB activation, but on the other hand, it may also reflect the extent of suppression of the NF-κB pathway. Thus, we focused on NF-κB2-luc2 transfected mice for drug inhibition studies. Pre-treatment with MLN120B (300 mg/kg orally at 16 and 1 hour prior to challenge) generated suppression of LPS-induced luciferase expression in the lungs when compared to the vehicle-control mice 4 hours after LPS challenge. Quantification of the lung signal showed an approximate 70% inhibition by MLN120B (Fig 5A,B). After imaging, mice were sacrificed and BAL fluid was harvested for cytokine analysis. Induction of MIP-1β, MCP-1, KC, IL-6 and TNFα was observed in BAL from LPS treated mice (Fig 5C). Significantly decreased levels of the pro-inflammatory chemokine MIP-1β and increased levels of the anti-inflammatory cytokine IL-10 were detected in the BAL of MLN120B pre-treated mice as opposed to LPS-treated positive controls.

### Suppression of NF-κB activation in the lungs by a glycogen synthase kinase beta TDZD-8

**Figure 6:**
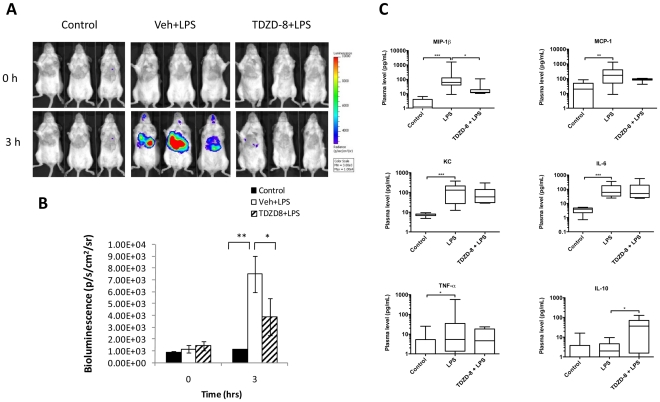
Effect of glycogen synthase kinase beta inhibition on NF-κB induction in vivo. NF-κB2-luc2 reporter transfected mice (n = 6 per group) were pre-dosed with either TDZD-8 (10 mg/kg, i.p.), or DMSO vehicle control 16 and 1-hour prior to LPS challenge. Mice were imaged immediately before LPS injection (T = 0) and at 3 hours post injection. Another group of mice was not treated with LPS and served as negative controls (n = 3). Bioluminescence images (A) and quantification of lung signals (B) are shown. Cytokines measured in bronchial lavage fluid as specified (C). Data presented as standard box and whisker plots, *p<0.05, **p<0.01, ***p<0.001 by Mann-Whitney U test.

As shown above (Fig 4), TDZD-8 is more potent than MLN120B in mitigating LPS induced NF-κB activation in Raw264.7 cells with all three reporters except NF-κB2-luc2. We examined its effect on NF-κB activation during LPS-induced lung inflammation in NF-κB2-luc2 transfected mice. Mice treated with LPS alone exhibited induction of bioluminescence signals in the lungs as expected. Pre-treatment of mice with 10 mg/kg of TDZD-8 (i.p.) totally abolished LPS induced signals in the lungs (Fig 6A, B). In addition, significantly decreased levels of the pro-inflammatory chemokine MIP-1β and increased levels of the anti-inflammatory cytokine IL-10 were detected in the BAL of TDZD-8 pre-treated mice as opposed to LPS-treated positive controls (Fig 6C).

## Discussion

The NF-κB transcription factor family plays a pivotal role in regulating innate inflammatory response underlying the pathogenesis of pulmonary diseases [9], [10]. We evaluated the utility of three NF-κB responsive reporters for monitoring NF-κB activation. When comparing the extent of induction under both in vitro and in vivo conditions, differences were observed. TNFAIP3-luc2 mediated the most robust luciferase induction in vivo, but showed the lowest induction in vitro among all three reporters. It is likely that the responsiveness of these reporters will be tissue and induction model dependent. Nonetheless, the difficulty of monitoring NF-κB activation in the lung non-invasively was overcome by transfecting mice with NF-κB reporters. We used intratracheal LPS delivery to demonstrate specific NF-κB induction in the lungs. Chronic exposure to LPS is reported to be associated with the development and/or progression of many types of lung diseases, including asthma, chronic bronchitis, and progressive irreversible airflow obstruction that are all characterized by chronic inflammatory processes in the lung. [4]. Thus our model will be useful for studying the NF-κB signaling pathway in these diseases.

Delivery of cationic polyethylenimine (PEI)/DNA complex caused mortality of the injected animal. It has been reported that pulmonary delivery of cationic lipid-DNA complexes can induce acute inflammation and tissue damage due to the presence of immunostimulatory CpG motifs within the plasmid DNA vector [24]. Thus, High NF-kB activity at day1 after transfection (Fig 2) was a manifestation of acute lung inflammation induced by the delivered DNA. Following the acute response, inflammatory NF-κB signal in the lungs declined to basal levels by two weeks. Our study also indicated that cationic PEI/DNA elicited toxicity was mouse strain dependent. When 50 µg of DNA was injected intravenously per animal, C57BL/6 albino mice showed an approximate 80% survival rate whereas none of the BALB/cJ mice survived. With lower quantities of DNA (25 µg), BALB/cJ mice exhibited a 100% survival rate (n = 2). Gene delivery to the respiratory system is of clinical interest for treatment of pulmonary diseases such as lung cancer, cystic fibrosis, and allergen-induced airway hyper-responsiveness [25]. Bioluminescence imaging is an ideal approach for evaluating gene delivery efficiency in animal models due to its superior sensitivity. In this study, we used an intravenous approach for gene delivery to lung tissues; however, recent studies demonstrated delivery of luciferase reporters as inhaled aerosols with sensitive detection of pulmonary luciferase activity in mice [26], [27]. A comparison of different agents and approaches for gene delivery efficiency will be explored in future studies, especially as gene therapy regimens are considered for clinical application.

Due to the pathophysiological complexity of chronic pulmonary diseases, efforts have been made to identify anti-inflammatory drugs that can attenuate the pro-inflammatory process at an early stage of disease. Hallmarks of the pathophysiology of lung inflammation are the release of multiple pro-inflammatory cytokines, reactive oxygen species, and infiltration of neutrophils into inflamed tissues. The NF-κB pathway plays a pivotal role in mediating this pathophysiologic development and constitutes a potential therapeutic strategy for inhibiting the release of inflammatory mediators. Establishment of imaging based animal models for tracking NF-κB activation in the lung enables high-throughput in vivo drug screening. As a proof of concept study, we tested the effect of two anti-inflammatory compounds on NF-κB activation in the lung. MLN120B is a potent and selective small molecule inhibitor of IKK-2, which is a predominant activator of NF-κB. Suppression of IKK-2 by MLN120B correlates with inhibition of IκBα phosphorylation and degradation in mouse tissues after the animals were given a single oral dose of 300 mg/kg [28]. With the same dose, we demonstrated that MLN120B significantly attenuated LPS induced NF-κB reporter activation in the lungs. We observed very little inhibitory effect on LPS induced NF-κB reporter activation in the lungs when a lower dose of 50 mg/kg MLN120B was used (result not shown). The MLN120B drug efficacy with different dosages resembles a recent study which showed that MLN120B had a robust inhibitory effect on arthritis development when dosed to mice orally twice a day at 60 mg/kg, but had very little effect at 10 or 30 mg/kg [29].

Involvement of GSK3ß in numerous intra-cellular signaling pathways underscores its etiological role in a number of diseases, including diabetes, Alzheimer's disease, bipolar disorder, and cancer [16]–[18]. A recent report showed that in innate immune cells, GSK3 inactivation augments anti-inflammatory cytokine production while concurrently suppressing the production of pro-inflammatory cytokines [30]. Ability of GSK-3ß to affect the NF-κB signaling pathway has been shown in a number of studies [31], [32]. GSK-3ß protects hepatocytes from TNF-induced apoptosis through p65 phosphorylation and upregulation of NF-κB transactivation [31]. In GSK-3ß gene-deleted cells, NF-κB activation induced by LPS, IL-1ß, or cigarette smoke condensate was completely suppressed, due to suppression of IκBα kinase activation and IκBα phosphorylation, ubiquitination, and degradation [19]. Animal studies have demonstrated that GSK3 inhibitors can suppress inflammation and protect the host from inflammation-mediated pathology and death. GSK inhibitors are being actively developed as drugs for the treatment of various disorders [33]. TDZD-8 is a potent selective inhibitor of GSK-3ß. In vivo studies have demonstrated that inhibition of GSK-3ß by TDZD-8 reduces the renal and liver dysfunction caused by both administration of endotoxin and peptidoglycan, and that this protective effect is due to inhibition of the phosphorylation of the NF-κB subunit [32]. Inhibition of GSK-3ß by TDZD-8 also attenuates carrageenan-induced lung injury, accompanied by suppression of IκBα degradation and P65 phosphorylation [34]. Using our NF-κB2-luc2 transfected mice, we demonstrated that TDZD-8 significantly suppressed NF-κB induction by LPS.

Significant induction of MIP-1β, MCP-1, KC, IL-6 and TNFα was observed in BAL during LPS induced lung inflammation. These pro-inflammatory cytokines act in concert to propagate inflammation [35]–[39] and elicit injury through the release of reactive oxygen species (ROS) and proteolytic enzymes [40]. The anti-inflammatory cytokine IL-10, can inhibit LPS-induced pro-inflammatory cytokines by inhibiting the NF-κB signaling pathway [41]. Thus, the observed effect of MLN120B and TDZD-8 in our study may be in part due to up regulation of IL-10. Indeed, in prior studies, the absence of endogenous IL-10 enhanced myeloperoxidase mediated oxidative stress and acute lung injury [35]. Interestingly, only the pro-inflammatory chemokine MIP-1β was down-regulated by MLN120B and TDZD-8. Prior studies suggest that of the CC chemokines, MIP-1β plays the most important role in intrapulmonary recruitment of neutrophils and in development of lung injury [42]. Collectively, our study shows that MLN120B and TDZD-8 exert anti-inflammatory effects associated with suppression of the NF-κB signaling pathway and the pro-inflammatory chemokine MIP-1β, while inducing the anti-inflammatory cytokine IL-10.

In summary, our in vivo gene delivery approach for monitoring NF-κB activation in the pulmonary tissue provides a sensitive noninvasive readout of inflammation kinetics. A multitude of activators and inhibitors concertedly regulate the NF-κB signaling pathway. Despite this complexity, the NF-κB2-luc2 based reporter system was able to detect a reduction in inflammation when different activators of this signaling pathway were inhibited. Our model may facilitate further pre-clinical characterization of new anti-inflammatory compounds for treating pulmonary diseases. In addition, targeted delivery of these NF-κB reporters to other tissues may also be a useful imaging approach for research in many other disease areas.

## Materials and Methods

### Reagents

Bacterial lipopolysaccharide (LPS, from *Salmonella abortus-equi*) and TDZD-8 were obtained from Sigma-Aldrich Chemical Co., (St. Louis, MO). Recombinant TNFα was obtained from R&D Systems (Minneapolis, MN), Lipofectamine LTX Transfection Reagent was obtained from Invitrogen, in vivo JetPEI DNA transfection reagent (Polyplus Transfection) was obtained from VWR (Brisbane, CA), and MLN120B was obtained from Chiesi Pharmaceutical (Marma, Italy).

### Cell based assay of NF-κB-luc reporters

We obtained NF-κB reporters from Switch Gear Genomics (Menlo Park, CA). The constructs contain the promoters of TNFAIP3, NF-κB2 or IL8 driving expression of the firefly luciferase (luc) reporter. Raw264.7 cells of 70% confluency were trypsinized and washed with PBS. Subsequently 1–3×10^7^ cells were suspended in 4 ml DMEM (without fetal calf serum or antibiotics). 50 µg of NF-κB -luc reporter DNA was mixed with 50 µl of Plus Reagent and incubated at room temperature for 5 minutes. Next, 100 µL of Lipofectamine LTX was added to the DNA mix and incubated at room temperature for 30 min. The DNA mixture was added to the cells together with 10 ml of media, and the mixture was transferred to a T75 flask, followed by 4 hour incubation at 37°C with shaking. The cells were then plated to a 96 well plate with 150 µl cells/well. Subsequently, 50 µl of 5X LPS solution and 50 µl of NF-κB inhibitor solutions (in DMEM growth media) were added to the plate and the cells were incubated overnight in a humidified incubator under a 5% CO2 atmosphere. After overnight incubation, the reporter activity was assayed through imaging with in vivo imaging system (IVIS®; Caliper Life Sciences., Alameda, CA) after adding 20 µl of 30 mg/ml luciferin to the each well.

### In vivo gene delivery

We used in vivo JetPEI (Polyplus Transfection) as a carrier for delivering DNA to the lung tissues. The DNA and JetPEI were formulated according to the manufacturer's instructions with a final N/P ratio of 7. Briefly, 50 µg of an NF-κB-luc reporter and 7 µl of JetPEI were each diluted into 200 µl with 5% glucose. The two solutions were then mixed and incubated for 15 minutes at room temperature. The entire mixture (400 µl) was injected intravenously into albino C57BL/6 mice and the expression of the NF-κB reporter was monitored through imaging with an IVIS® imaging system.

### In vivo bioluminescence imaging

Mice were anesthetized with isoflurane and imaged for 5 minutes following intraperitoneal injection of 150 mg/kg luciferin. Imaging was performed with an IVIS Spectrum. Bioluminescent signals were quantified using Living Image® software (Caliper Life Sciences., Alameda, CA.).

### Acute pulmonary Inflammation and the effect of anti-inflammation compounds

LPS was dissolved in sterile PBS to a concentration of 10 mg/ml. LPS (in 50 µl total volume) was delivered to mice intratracheally with a 22-gauge intubator. The final dose of LPS was 1 mg/kg. To perform a dosage study with TNFα, the cytokine solution was diluted in PBS to concentrations of 0.5, 1, or 2 µg in a 100 µl volume and the solutions were delivered to mice intravenously. Induction of NF-κB reporters was monitored through imaging with IVIS following i.p. injection of luciferin at specific time points. To study the effect of anti-inflammatory compounds, mice were administered with 4-benzyl-2-methyl-1,2,4-thiadiazolidine-3,5-dione (TDZD-8) (in DMSO, 10 mg/kg, i.p.), or MLN120B (in 0.5% methylcellulose, 300 mg/kg, p.o.) at 16 hours and 1 hour prior to intratracheal delivery of LPS.

### Harvesting bronchoalveolar lavage fluid and cytokine analysis

Bronchoalveolar lavage (BAL) was collected as previously described [38]. Briefly, mouse tracheas were cannulated with an 18-gauge angiocath (Becton Dickinson). The lungs were instilled with 1 ml of sterile saline 4 times and a total of 3–4 ml lavage fluid was collected. After centrifugation at 500 *g* for 10 min, the BAL fluid was collected and frozen at −80°C until measurement of interferon (IFN)-γ, interleukin (IL)-6, IL-10, tumor necrosis factor (TNF)α, monocyte chemoattractant protein (MCP)-1, macrophage inflammatory protein (MIP)-1β, and keratinocyte-derived chemokine/CXCL1 (KC) quantified using the Cytometric Bead Array Mouse Flex Kit (BD Biosciences; San Jose, CA). The cell pellet was resuspended in 1 ml of 1% BSA in sterile physiological saline. Total cell counts were determined using a grid hemocytometer. Serum was obtained by cardiac puncture. Lungs were harvested by resection, and tissues were immediately stored in 10% formalin.

### Statistical analysis

Significance between two means was calculated using a two-tailed Mann-Whitney U test. A *p* value of <0.05 was considered significant. Statistical analyses were performed using Prism 5 (GraphPad Software, Inc.; La Jolla, CA).
